# Posttransplant Lymphoproliferative Disorder Presenting as Testicular Lymphoma in a Kidney Transplant Recipient: A Case Report and Review of the Literature

**DOI:** 10.1155/2018/9787093

**Published:** 2018-02-14

**Authors:** Steve Omoruyi Obanor, Michelle Gruttadauria, Kayla Applebaum, Mohammad Eskandari, Michelle Lieberman Lubetzky, Stuart Greenstein

**Affiliations:** ^1^Montefiore Einstein Center for Transplantation, Montefiore Medical Center, Albert Einstein College of Medicine, Bronx, NY, USA; ^2^Department of Internal Medicine, Maimonides Medical Center, Brooklyn, NY, USA; ^3^Albert Einstein College of Medicine, Bronx, NY, USA; ^4^Department of Pathology, Montefiore Medical Center, Albert Einstein College of Medicine, Bronx, NY, USA

## Abstract

Posttransplant lymphoproliferative disorder (PTLD) is a malignancy caused by the immunosuppression that occurs after transplantation. It is primarily a nodal lesion but frequently it involves extranodal masses. Treatment is usually by reducing immunosuppressive therapy. Testicular lymphoma as PTLD is notably rare in documented literature and there is limited evidence of definitive treatment guidelines. This manuscript describes a patient who developed diffuse large B-cell lymphoma of his right testis one year following kidney transplantation. A diagnosis of PTLD was made and treatment with rituximab, locoregional radiotherapy, and intrathecal methotrexate in addition to the standard reduction of immunosuppression resulted in complete remission until now. We submit this case along with literature review of similar cases in the past and a review of specific peculiarities of our case with emphasis on our treatment plan to further the understanding of this diversiform disease.

## 1. Introduction

Posttransplant lymphoproliferative disorder (PTLD) is a heterogeneous group of lesions that can develop in patients receiving chronic immunosuppression after solid organ transplantation [1]. The clear majority of these are proliferations of B-cell origin, triggered by Epstein-Barr virus (EBV) infection [2]; this can either be a primary infection after transplant in a seronegative recipient or reactivation of latent EBV infection in a seropositive recipient, the former posing higher risk of development of PTLD [3]. They present with symptoms of infectious mononucleosis or with fever and localized or disseminated lymphoproliferation involving the lymph nodes and extranodal sites like the liver, lungs, kidney, bone marrow, central nervous system, or small intestine [4]. Although PTLD has increased extranodal involvement when compared with lymphoma in the general population [5], cases involving extranodal masses in the testes have very sparse representation in current literature. We report an unusual finding of PTLD in a kidney transplant patient presenting as lymphoma of the right testis. We include a comparison of this form of PTLD with primary testicular lymphoma in the immunocompetent patient and review literature considering the role of EBV in carcinogenesis.

## 2. Materials and Methods: The Case Report

A 68-year-old Hispanic male (postrenal transplant) presented to our follow-up clinic in December 2013 with a 2-week history of painless right testicular swelling. He denied trauma. There was no associated history of fever, weight loss, anorexia, night sweats, or urinary symptoms.

His past medical history was significant for morbid obesity, diabetes, lone atrial fibrillation, and end-stage renal disease secondary to hypertensive nephrosclerosis for which he underwent a deceased donor renal transplant one year prior to presentation. He received a Public Health Service and Centers for Disease Control (PHS/CDC) high-risk kidney. EBV IgG and IgM and Cytomegalovirus (CMV) IgG and IgM of donor were negative. For the recipient, EBV IgG and CMV IgG were positive; however, EBV viral capsid antigen IgM, EBV DNA PCR, and CMV IgM were negative. Calculated Panel Reactive Antibody was 0. There were no donor-specific antigens (DSA). T&B cell flow crossmatches were negative. He received induction with basiliximab and maintenance immunosuppression with tacrolimus, mycophenolic acid, and prednisone. He had no episodes of graft rejection. He also had a history of Polyomavirus BK viremia, which necessitated a mycophenolate dose reduction (1000 mg twice a day (BID) to 500 mg BID). He was a former smoker but quit over 20 years ago and his mother is said to have had an unnamed cancer.

Initial management was with outpatient urology consult. Physical examination revealed an enlarged indurated right testicle with normal lie, firm-hard, nontender, nonfluctuant, and no regional or generalized lymphadenopathy. Left hemiscrotum was grossly normal. Blood and sonographic tests were ordered and he was asked to follow up in a month.

Serum tumor markers, alpha fetoprotein, beta human chorionic gonadotropin, prostate-specific antigen, and lactate dehydrogenase were all within normal limits. EBV and CMV serologies remained unchanged from pretransplant values. HIV was negative.

**Figure 1:**
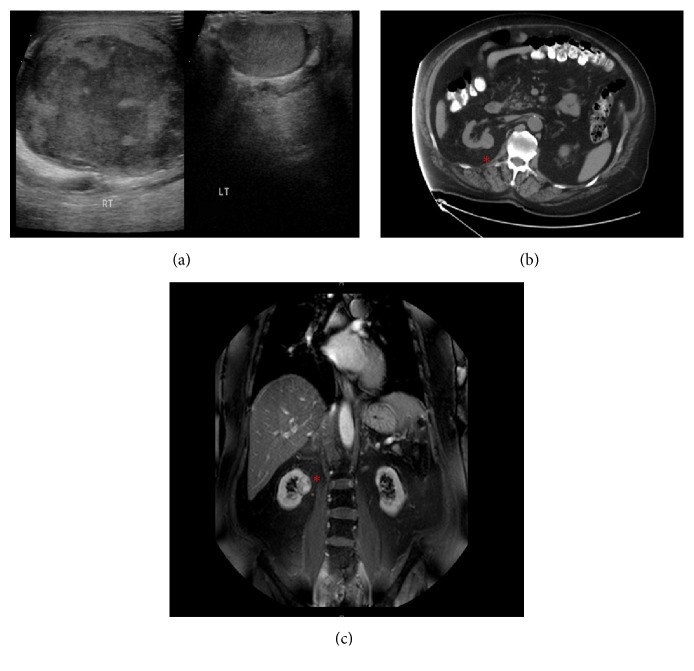
(a) Sonogram of both testes: sagittal view showing grossly enlarged right testis in comparison with the left. (b) T2-weighted MRI of the abdomen with contrast, transverse, and (c) sagittal views; renal cell carcinoma (red asterisk) noted on the right native kidney.

Doppler sonographic findings noted a markedly abnormal right testis, which was enlarged and nearly completely replaced by a zone or mass of heterogeneously decreased echogenicity and little if any internal vascular flow, suggestive of a subacute infarct or a hypovascular tumor (Figure 1(a)).

Prior to his next follow-up visit, however, he presented to the Emergency Department complaining of sudden sharp, nonradiating right testicular pain without fever or urinary symptoms. Noncontrast CT revealed an incidental, solitary, 2.3 × 2.1 cm, partially exophytic upper pole lesion on the right native kidney, suspicious for neoplasm; there was no abdominal or pelvic lymphadenopathy. Magnetic resonance imaging with contrast showed a small enhancing right renal nodule suspicious for cortical malignancy but there was no evidence of metastatic disease (Figures 1(b) and 1(c)). Mycophenolic acid was withheld due to concern for undetected malignancy.

In January 2014, he underwent a right total nephrectomy and right radical orchiectomy.

**Figure 2:**
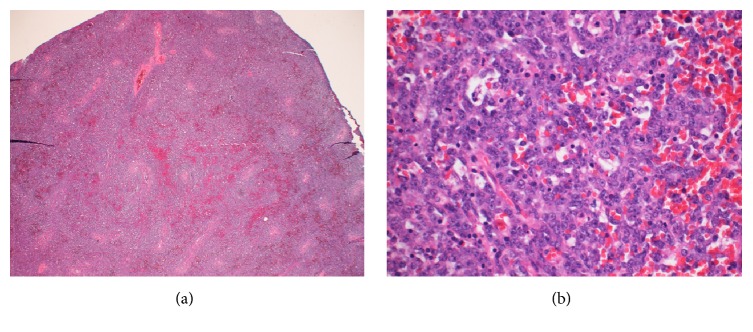
Section of the right testis stained with hematoxylin and eosin. (a) Low power (2x) shows diffuse infiltration of the testis by large neoplastic cells. There is geographic necrosis. (b) Higher magnification (40x) shows the large cells with irregular nuclei, vesicular chromatin, and prominent nucleoli. Occasional mitoses and apoptotic bodies present.

**Figure 3:**
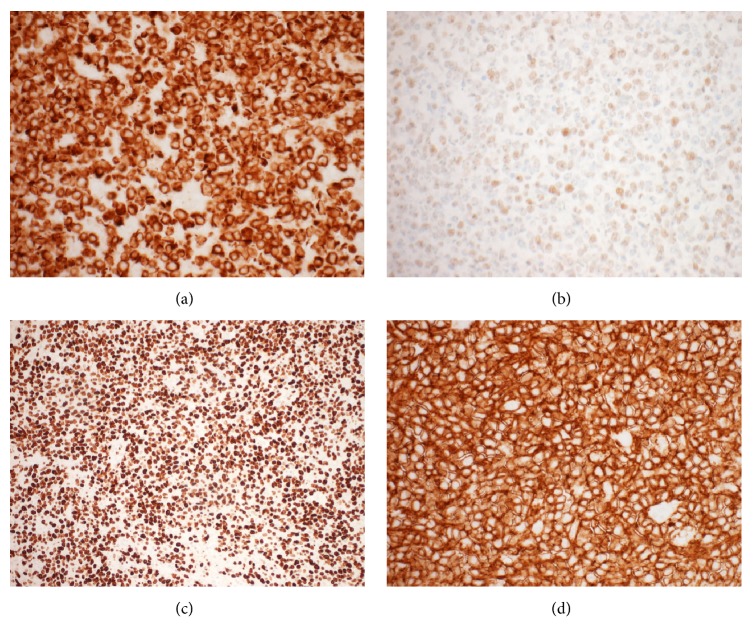
Histopathological findings of the monomorphic posttransplant lymphoproliferative disorder; diffuse large B-cell lymphoma of the right testis. Immunophenotyping, via immunohistochemistry, revealed neoplastic cells positive for Bcl-2 (a), Bcl-6 (b), Ki-67 (proliferation index > 90%) (c), and CD20 (d). Magnification for all panels: 40x.

Pathological analysis established the diagnosis of renal cell carcinoma (RCC) and clear cell type (Fuhrman Nuclear grade 2). The lesion was 2.5 cm in its greatest dimension and unifocal and was limited to the kidney with all margins clear. Histological analysis of the right testis revealed a monomorphic posttransplant lymphoproliferative disorder: diffuse large B-cell lymphoma (DLBCL) (Figure 2). Immunohistochemistry of the abnormal B-cells was positive for CD20, Bcl-2, Bcl-6, MUM1, Ki-67 (at least 90%), and EBV latent membrane protein 1 (EBV LMP-1) (Figure 3). The cells were negative for CD3, CD5, CD10, c-Myc, CD30, and HHV8. Fluorescent in situ hybridization and molecular studies were not performed.

Upon diagnosis of lymphoma, he had reduction of immunosuppression (mycophenolic acid was withheld) and was maintained on low-dose tacrolimus (0.5 mg q12 h) and prednisone (5 mg daily). Five days prior to diagnosis of lymphoma, the patient's tacrolimus level was 7 nanograms/mL. Cerebrospinal fluid (CSF) cytology, CSF flow cytometry, and CT guided bone marrow biopsy all showed no evidence of clonal B-cell expansion. PET/CT from base of skull to mid-thigh showed no evidence of metastatic disease which further characterized the disease as Stage IE.

Considering the rare nature of posttransplant testicular lymphoma, two oncologic opinions were sought. The initial recommendation was rituximab, cyclophosphamide, hydroxydaunorubicin, and prednisone (R-CHOP) regimen, in combination with intrathecal methotrexate and radiation to the contralateral testes. However, given his advanced age and immunosuppressed state, an alternative opinion to receive the above treatment without the CHOP regimen was recommended.

He went on to receive intrathecal methotrexate, 4 courses of rituximab, and radiation to the contralateral testes, which was completed in July 2014. Within 6 months, he achieved complete remission while maintaining excellent allograft function. His current creatinine is 1.2, although he has developed de novo DSA in the setting of reduced immunosuppression. He has no proteinuria and this is being followed closely as well as regular follow-up with oncology, with recurrence surveillance provided by regular PET/CT scans per oncology protocols.

## 3. Discussion

The incidence of PTLD in adult kidney transplant recipients ranges from 1 to 2.3% [6] and although immunosuppression, as can be seen after a transplant, puts the male patient at a 20 to 50 times greater risk of development of testicular neoplasm [7, 8], testicular lymphoma is still exceedingly rare in this population. A retrospective study analyzed malignant testicular neoplasms in immunocompromised (AIDS and posttransplant) patients over a period of 20 years. This study reviewed histopathology of testicular tumors, patient ages at presentation, disease stage at presentation, management schemes, type and incidence of adverse effect of therapy, and/or outcome after therapy. The tumors found were germ cell tumors (seminomas and nonseminomas, with equal prevalence) and lymphomas. However, no lymphomas were noted among posttransplant patients [9]. Prior to this case, only two solid organ recipients had been reported to develop testicular lymphoma after transplant: a 52-year-old with a primary occurrence of aggressive large cell CD45+ epididymal lymphoma seven years after transplant treated with reduction in immunosuppression and CHOP [10] and an 8-year-old heart transplant patient with CD20+, EBV+ polymorphic PTLD of the testicle six years after transplant treated with rituximab and autologous EBV-specific cytotoxic T lymphocytes [11].

Organ transplant recipients maintained on chronic immunosuppressive therapy have a heightened chance of developing de novo cancer within the first few years of transplantation [12]. However, on presentation with initial symptoms, our patient seemed to have a low risk of developing PTLD and presence of latent EBV infection as opposed to a primary one; the type of organ transplanted was the kidney, being the least likely to result in PTLD; induction therapy was by basiliximab as opposed to muromonab-CD3 (OKT3) or thymoglobulin; he had no prior rejection episodes and was neither CMV nor Hepatitis C Virus (HCV) positive. This stresses the need to have a high index of suspicion of PTLD. In addition to testicular lymphoma, he also developed a right renal cell carcinoma, an occurrence which can be attributed to his immunosuppressed state, morbid obesity, or even his family history of cancer. Several studies, however, have described the possible role of EBV in the etiopathogenesis of tumors after transplant. Lee et al. studied three children in whom smooth-muscle tumors developed at varying periods of time after transplant [13]. They found that a single form of EBV DNA was present in each tumor, defining them as neoplasia. Also, on analysis of EBV DNA in the tumors, features akin to those found in PTLD including unique EBV DNA episomes (EBV small RNA, EBER, and EBV nuclear antigen-2 (EBNA-2)) were noted. These unique episomes showed that they were present before the clonal population was derived, strongly supporting a causative role for EBV in the development of smooth-muscle tumors after transplantation. Shimakage et al. have several studies reporting the role of EBV in the pathogenesis of many human cancers. One of their studies [14] attempted to investigate EBV expression in renal cell carcinoma with the aim of inferring a causal relationship. Formalin-fixed paraffin-embedded tissue samples from nine patients with RCC and two patients with nephroblastoma were subjected to mRNA in situ hybridization and indirect immunofluorescence staining. Their results indicated that mRNA and proteins of EBV were expressed in all RCC and nephroblastoma tissues, suggesting an oncogenic and tumor progressive role of EBV in these conditions. This was in keeping with a prior study [15] on EBNA-2 transgenic mice, 90% of which developed kidney adenocarcinoma (immunohistochemistry demonstrated nuclear expression of EBNA-2 in hyperplastic tubular cells and tumor cells). Shimakage et al. also observed that EBV expression was more commonly present in papillary and clear cell RCC than chromophobe cell RCC (chromophobe cell RCC described to be less malignant than other RCC). Furthermore, EBV was expressed at higher rates in high-grade RCC than in low-grade RCC. These both suggested that EBV expression correlated with RCC malignancy. It is possible therefore to postulate that both neoplastic occurrences in our patient resulted as a consequence of EBV infection after transplant.

Primary testicular lymphoma in an immunocompetent patient is an aggressive form of extranodal lymphoma. Though rare, it is the most common testicular malignancy in men aged above 60 years (median age at diagnosis is 66 to 68 years). Our patient was 68 years old at presentation of symptoms and had atypical transplant serologic markers for PTLD. He was EBV IgG+ with an EBV− donor kidney. It was therefore paramount to distinguish his lymphoma as a PTLD in order to decide upon appropriate treatment. The presence of EBV LMP-1 (latent membrane protein 1) in the tissue sufficiently establishes this diagnosis. Serologic markers of EBV infection have also been used by some clinicians in making the diagnosis of PTLD, serum EBV DNA PCR (EBV DNAemia) being one of the most widely studied. Although EBV DNAemia has been suggested as a means of surveillance for suspected PTLD cases and monitoring established cases, several studies have shown its use to be debatable [16]. Initially thought to have 100% sensitivity and specificity, EBV DNAemia has now been found to be relatively less sensitive though maintaining fairly high specificity for the detection of PTLD [17–19]. EBV DNA was negative in peripheral blood samples in our patient; possible reasons for this could be the fact that the PTLD was not driven by EBV or the effect of EBV infection on lymphoproliferation was localized [20]. Since our case showed EBV association histologically, the latter option is highly probable. Several studies show similar cases of localized PTLD failing to provoke a corresponding EBV DNAemia [20, 21]. One of such studies involves a case of isolated CNS lymphoma after allogenic hematopoietic stem cell transplant, in which EBV DNA load was elevated in the cerebrospinal fluid but was not detected in the peripheral blood [22]. In this case, it is important to note the special characteristics shared by both the CNS and the testes, as both are immune privileged sites, with tight junctions which isolate these cells from peripheral blood. This may play a role in negative EBV DNAemia seen in localized forms of PTLD.

The current international standard of care for primary testicular lymphoma irrespective of immune status is R-CHOP every 21 days with intrathecal methotrexate and locoregional radiation therapy [23]. In the international trial, intrathecal rather than systemic CNS prophylaxis was used as it is better tolerated by elderly patients than aggressive high-dose systemic prophylaxis [23]. There are no specific guidelines for testicular PTLD due to the rarity of its presentation. After consultations with 2 independent oncology services, we opted out of systemic chemotherapy, primarily because of the patient's advanced age and comorbidities. Local consensus studies also suggest that systemic chemotherapy is most useful in patients with monomorphic histology, a large mass lesion, fulminant PTLD, or evidence of graft rejection [24]. Furthermore, existing literature pertaining to the management of primary testicular lymphoma was reviewed, which found that adverse prognostic factors [25] include age above 70 years, advanced stage, presence of B symptoms, >1 extranodal site, involvement of extranodal sites other than testis, tumor diameter > 10 cm, elevated lactate dehydrogenase, elevated B2-microglobulin, hypoalbuminemia, and involvement of the left testes, all of which were negative in our patient. This also helped inform the decision for treatment without CHOP therapy. Estimated CNS involvement in primary testicular lymphoma has been reported in smaller series as up to 44% [25]. This risk has been noted, in a large retrospective study [26], to be higher in extranodal DLBCL as opposed to their nodal counterparts. Given CD20+ status of the lesion and the above considerations, treatment with rituximab, intrathecal methotrexate, and locoregional radiotherapy was agreed upon. Although this treatment course resulted in a positive outcome in this patient, strong conclusions cannot be made with this limited evidence of success and short follow-up period. It instead highlights the need for further investigation and discussion.

## 4. Conclusion

In summary, we report a unique manifestation of PTLD presenting as a testicular lymphoma, still a rare form of extranodal PTLD. Standard management of PTLD is usually with reduction of immunosuppression alone; however, given the aggressive nature of the lymphoma, we came up with a unique combination of surgical excision, reduction of immunosuppression, rituximab, intrathecal methotrexate, and radiotherapy. With this strategy, complete remission and a disease-free state until now were achieved.
